# The value of cardiopulmonary comorbidity in patients with acute large vessel occlusion stroke undergoing endovascular thrombectomy: a retrospective, observational cohort study

**DOI:** 10.1186/s12883-024-03660-w

**Published:** 2024-05-07

**Authors:** Jiarui Wang, Yongqiang Cui, Xiangkai Kong, Bin Du, Tian Lin, Xiaoyun Zhang, Dongxu Lu, Li Liu, Juan Du

**Affiliations:** 1 PLA 306 Clinical College, Anhui Medical University, Beijing, China; 2 The Fifth Medical college, Anhui Medical University, Beijing, China; 3 Department of Neurology, PLA Strategic Support Force Characteristics Medical Center, 9 Anxiangbeili Rd, Beijing, 100086 China

**Keywords:** Cerebral infarction, Acute ischemic stroke, Endovascular thrombectomy, Prognosis, Comorbidity

## Abstract

**Background:**

Chronic lung and heart diseases are more likely to lead an intensive end point after stroke onset. We aimed to investigate characteristics and outcomes of endovascular thrombectomy (EVT) in patients with acute large vessel occlusion stroke (ALVOS) and identify the role of comorbid chronic cardiopulmonary diseases in ALVOS pathogenesis.

**Methods:**

In this single-center retrospective study, 191 consecutive patients who underwent EVT due to large vessel occlusion stroke in neurological intensive care unit were included. The chronic cardiopulmonary comorbidities and several conventional stroke risk factors were assessed. The primary efficacy outcome was functional independence (defined as a mRS of 0 to 2) at day 90. The primary safety outcomes were death within 90 days and the occurrence of symptomatic intracranial hemorrhage(sICH). Univariate analysis was applied to evaluate the relationship between factors and clinical outcomes, and logistic regression model were developed to predict the prognosis of ALVOS.

**Results:**

Endovascular therapy in ALVOS patients with chronic cardiopulmonary diseases, as compared with those without comorbidity, was associated with an unfavorable shift in the NHISS 24 h after EVT [8(4,15.25) versus 12(7.5,18.5), *P* = 0.005] and the lower percentage of patients who were functionally independent at 90 days, defined as a score on the modified Rankin scale of 0 to 2 (51.6% versus 25.4%, *P* = 0.000). There was no significant between-group difference in the frequency of mortality (12.1% versus 14.9%, *P* = 0.580) and symptomatic intracranial hemorrhage (13.7% versus 19.4%, *P* = 0.302) or of serious adverse events. Moreover, a prediction model showed that existence of cardiopulmonary comorbidities (OR = 0.456, 95%CI 0.209 to 0.992, *P* = 0.048) was independently associated with functional independence at day 90.

**Conclusions:**

EVT was safe in ALVOS patients with chronic cardiopulmonary diseases, whereas the unfavorable outcomes were achieved in such patients. Moreover, cardiopulmonary comorbidity had certain clinical predictive value for worse stroke prognosis.

**Supplementary Information:**

The online version contains supplementary material available at 10.1186/s12883-024-03660-w.

## Background

Acute ischemic stroke (AIS) is a central nervous system (CNS) disease leading the second cause of death worldwide and is proved to be influenced by many factors [1]. Although limited by treatment time window and classification of cerebral infarction, the efficacy and safety of endovascular thrombectomy (EVT) for eligible patients with acute large vessel occlusion stroke (ALVOS) have been fully confirmed by positive results of large randomized clinical trials in recent years [2–4]. Treatment futility criteria for EVT—characteristics defining patients’ subgroups that do not benefit from EVT—still remain elusive [5].

Recent data suggest that distinctive factors [6–9] may impact functional outcome in ALVOS patients. Chronic lung and heart diseases cause discomfort in activities of daily living (ADLs) or exercise [10] and are more likely to lead an intensive end point after stroke onset [11, 12]. Previous studies have focused on the impact of comorbidity on stroke, but none have considered whether comorbidity affect endovascular therapy and it is uncertain whether EVT could help retard functional deterioration. A better understanding of the association of comorbidity with stroke severity, intraoperative manipulation and outcomes is therefore required to understand apparent associations with poststroke functional independence. Therefore, our study was designed to test the hypothesis that cardiopulmonary comorbidities would lead to worse neurological functional outcomes by comparing the characteristics and outcomes between patients who underwent endovascular therapy with and without cardiopulmonary comorbidities.

## Methods

### Participant

This study was a retrospective analysis. We enrolled patients with anterior or posterior circulation LVOS who underwent EVT from January 2018 to September 2022 in our stroke center. The research protocol was reviewed and approved by the Ethics Committee (approval number: K2023-021-01). Written informed consent was obtained from all patients or their legal representatives. We have adhered to the STROBE statement (Supplementary material).

**Fig. 1:**
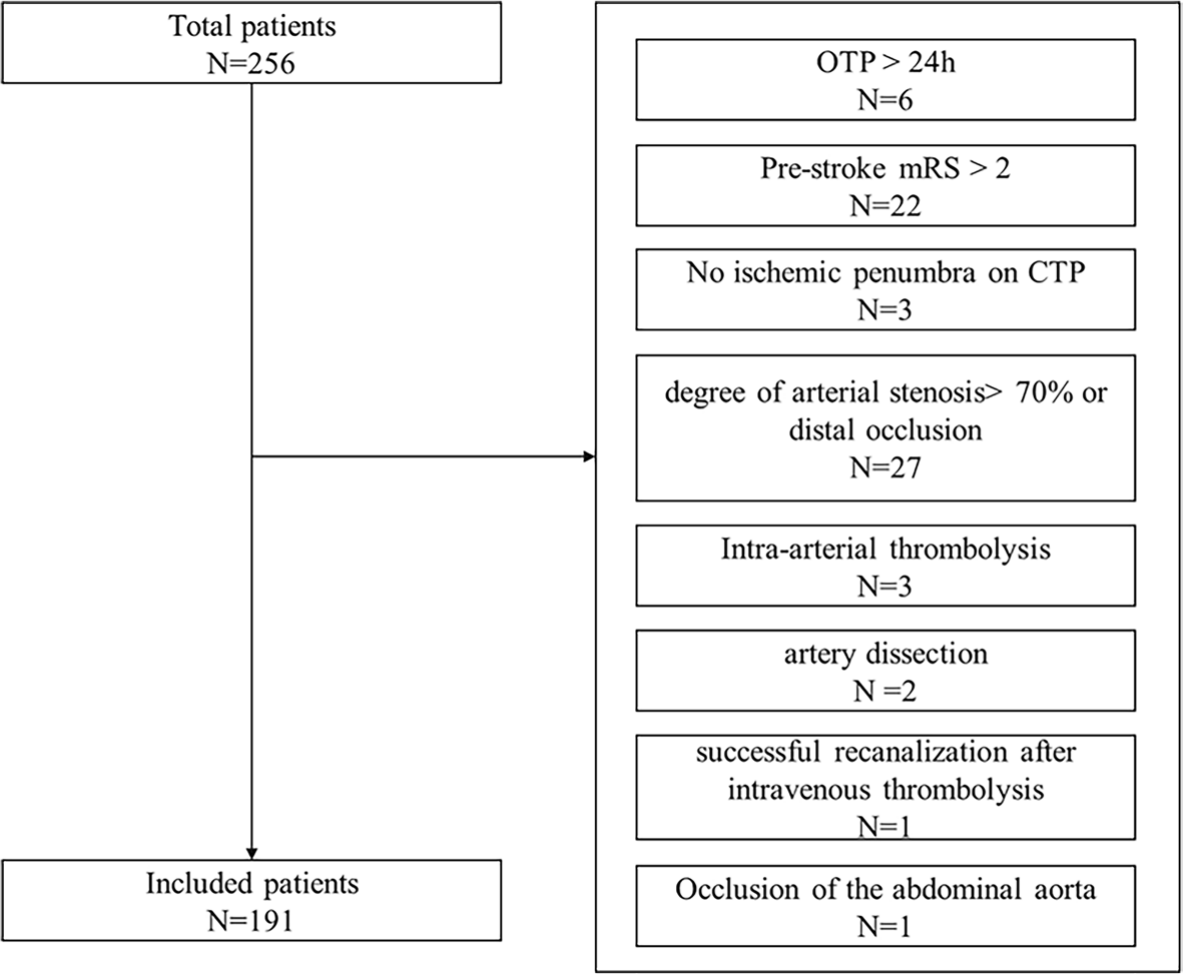
Flow chart of the inclusion of the study population. mRS = modified Rankin Scale, OTP = symptoms onset to groin puncture time

Patients were eligible if they met the following inclusion criteria. (a) Signs and symptoms consistent with the diagnosis of an acute ischemic stroke according to the criteria of Chinese guidelines for diagnosis and treatment of acute ischemic stroke 2018 [13]. (b) Age ≥ 18 years. (c) Endovascular therapy can be initiated (femoral puncture) in 24 h of stroke onset. Stroke onset is defined as the time the patient was last known to be at their neurologic baseline. (d) Occlusion of the internal carotid artery, proximal segment of the middle cerebral artery (M1/M2), the anterior cerebral artery (A1/ A2), or basilar artery (BA) can be confirmed by digital subtraction angiography (DSA) or computed tomographic angiography (CTA). The exclusion criteria includes: (a) intracranial hemorrhage; (b) massive brain infarction (anterior circulation infarction > 1/2 MCA territory, posterior circulation infarction > 2/3 Pontine or midbrain volume); (c) known allergy to iodine that precludes an endovascular procedure; (d) known hereditary or acquired hemorrhagic diathesis, coagulation factor deficiency; (e) previous severe disability [modified Rankin Score (mRS) [14] ≥ 3]; (f) severe renal failure. The treatment protocol and methods have been published before [15]. The flow chart of the inclusion of the study population is displayed in Fig. 1.

### Clinical assessment

The demographics characteristics and medical history of all patients were collected and recorded. Chronic cardiopulmonary comorbidity is a clear diagnosis of various cardiac diseases [rheumatic heart disease or various cardiomyopathy or coronary atherosclerotic heart disease (acute coronary syndrome or after coronary artery stent implantation or coronary artery bypass grafting)] with a New York Heart Association (NYHA) cardiac function grade III ~ IV or cardiac ultrasound showing left ventricular ejection fraction (LVEF) less than 45%; and/or a clear diagnosis of chronic obstructive pulmonary disease or pulmonary heart disease, with type I or type II respiratory failure before the stroke onset. Other clinical assessments were performed at baseline; assessments included the score on the modified Rankin scale (mRS) [14], the National Institutes of Health Stroke Scale (NIHSS) [16], the Trial of ORG 10,172 in Acute Stroke Treatment (TOAST) classification [17] and ASPECTS [18] or pc-ASPECTS [19]. The treatment-related information was recorded by a neurointerventionalist during thrombectomy therapy, including intravenous thrombolysis, occlusion site, tandem lesion, stroke onset to door time(ODT), door to puncture time (DPT), puncture to reperfusion time (PRT), anesthesia type, the thrombectomy approach (stent-retrievers or suction thrombectomy systems), rescue therapy (balloon dilation or stent implantation), times of pass through, modified thrombolysis in cerebral infarction (mTICI) score [20].

### Outcome

The primary efficacy outcome was functional independence (defined as a score on the modified Rankin scale of 0 to 2) at day 90. The secondary efficacy outcomes were the NIHSS right after and 24 h after endovascular therapy. The primary safety outcomes were death within 90 days and the occurrence of symptomatic intracranial hemorrhage(sICH), defined as an increase of at least 4 points in the NIHSS score that was associated with parenchymal, subarachnoid or intraventricular hemorrhage on CT imaging within 36 h after thrombectomy. The outcomes were collected by telephone or outpatient consultation at day 90 after EVT.

### Treatment

Patients with 6 to 24 h of onset were scanned by CT perfusion (CTP), and evaluated whether there was mismatch area. Anesthesia type depended on the situation, and femoral artery puncture was performed after anesthesia. Aspiration thrombectomy or stent-retriever thrombectomy was performed with approved thrombectomy device, at the discretion of the neurointerventionalist. If the thrombectomy did not successfully recanalize after 4 operations, balloon angioplasty or stent implantation could be used for rescue therapy. If the arteries were still blocked after rescue therapy, the treatment was considered to be failed. Permanent stenting and ballon angioplasty could be combined. All patients were admitted to neurological intensive care unit (NICU) for at least 72 h after the operation. Other perioperative medical therapy was carried out according to the guidelines [13].

### Statistical analysis

Python 3.10 software was used for all the statistical analysis. The Kolmogorov-Smirnov method was used to test the normal distribution of the continuous variables. Then the mean ± sd of the measurement data was used for the normal distribution, and the student test was used for the comparison between the groups. Metrical data that did not conform to normal distribution was expressed as median (interquartile range) [M (P25, P75)]; and Mann-Whitney U test was used for comparison between groups. The Categorical variables were expressed by the number of cases and percentage (%), and the comparison between groups was carried out by Chi-square test or Fisher’s exact test as appropriate. Statistically significant differences were defined as *P* < 0.05. Moreover, the prognosis model for primary efficacy outcomes was calculated by logistic regression, in which the variables were tested in the univariate analysis. Statistically significant differences were defined as *P* < 0.1 in this case. And due to the unbalanced outcomes, the research participants were stratified randomly divided into a training set and an internal validation set at a ratio of 7:3 to better the logistic regression model. A receiver operating characteristic (ROC) curve was drawn, and the area under the curve (AUC) was calculated to evaluate the predictive efficacy of the logistic regression model.

## Result

### General characteristic

A total of 191 ALVOS patients who received EVT were included in this study. The median age was 70(59, 80) years. Among them, there were 67 patients with cardiopulmonary comorbidity (CC group) and 124 patients with non-cardiopulmonary comorbidity (NCC group). The demographics and baselines characteristics of the overall patients are shown in Table 1. The characteristics were not balanced. Patients with cardiopulmonary complications were more likely to be female (27.4% versus 56.7%, *P* = 0. 000), older [67(57, 77) versus 76 (67.5, 82.5), *P* = 0.000], and to have atrial fibrillation (35.5% versus 71.4%, *P* = 0. 000) and coronary heart disease (14.5% versus 50.7%, *P* = 0. 000). Unexpectedly, the CC group was significantly less likely to smoke (24.2% versus 10.4%, *P* = 0. 022). There was difference in Toast classification of cerebral infarction between the two groups (*P* = 0.000). Cardioembolism (46.8%) and large-artery atherosclerosis (43.5%) were the main causes in the NCC group, while large-artery atherosclerosis (80.6%) was the main cause in the CC group. The NIHSS scores of the whole cohort at admission ranged from 0 to 40, with an average of 17.53 ± 7.08. There was no difference in baseline NIHSS scores (*P* = 0.293) and ASPECT (*P* = 0.073) scores. Before stroke onset, the distribution of mRS scores had a significant difference between the two groups (*P* = 0.023). Patients with cardiopulmonary complications were less likely to have pre-stroke mRS score at 0 (82.3% versus 67.2%).

**Table 1:** Demographics and baseline characteristics stratified by cardiopulmonary comorbidities

Characteristics	All patients (*n* = 191)	NCC group (*n* = 124)	CC group (*n* = 67)	Test value	*P*-value
Age, median(IQR)	70 (59,80)	67 (57,77)	76 (67.5,82.5)	2782.000^b^	0.000^*^
Male, n(%)	119 (62.3%)	90(72.6%)	29 (43.3%)	15.896^c^	0.000^*^
Medical history, n(%)					
Ischemic stroke	51 (26.7%)	29 (23.4%)	22 (32.8%)	1.984^c^	0.159
Diabetes mellitus	75 (39.3%)	46 (37.1%)	29 (43.3%)	0.698^c^	0.403
Hypertension	129 (67.5%)	82 (66.1%)	47 (70.1%)	0.321^c^	0.571
Hyperlipidemia	74 (38.7%)	42 (33.9%)	32 (47.8%)	3.536^c^	0.060
Atrial fibrillation	92 (48.2%)	44 (35.5%)	48 (71.4%)	22.778^c^	0.000^*^
Coronary heart disease	52 (27.2%)	18 (14.5%)	34 (50.7%)	28.817^c^	0.000^*^
Smoking	37 (19.4%)	30 (24.2%)	7 (10.4%)	5.262^c^	0.022^*^
Drinking	19 (9.9%)	16 (12.9%)	3 (4.5%)	3.447^c^	0.063
TOAST classification, n(%)				24.728^c^	0.000^*^
Cardioembolism	70 (36.6%)	58 (46.8%)	12 (17.9%)		
Large-artery atherosclerosis	108 (56.5%)	54 (43.5%)	54 (80.6%)		
Other	13 (6.8%)	12 (9.7%)	1 (1.5%)		
Baseline NIHSS, mean ± SD	17.53 ± 7.08	17.14 ± 7.44	18.27 ± 6.35	-1.054^a^	0.293
Pre-stroke mRS score, n(%)				6.002 ^c^	0.0497^*^
0	147 (77.0%)	102 (82.3%)	45 (67.2%)		
1	26 (13.6%)	14 (11.3%)	12 (17.9%)		
2	18 (9.4%)	8 (6.5%)	10 (14.9%)		
ASPECTS	8.52 ± 1.67	8.68 ± 1.56	8.22 ± 1.82	1.802^a^	0.073

*IQR* Interquartile range, *SD *Standard deviation, *TOAST *The Trial of ORG 10,172 in Acute Stroke Treatment, *NIHSS *The National Institutes of Health Stroke Scale, *mRS *Modified Rankin Scale, *ASPECTS *Alberta stroke program early CT score or pc-ASPECTS

^*^ for *P* < 0.05

^a^ for t-value, ^b^for Z-value, ^c^ for χ^2^ value

### Clinical characteristic

The clinical characteristics of the overall patients are shown in Table 2. The times of pass through was 2(1, 4) in the CC group and more than 1 (1, 3) in the NCC group, which were significantly different (*P* = 0. 001). The proportion of balloon angioplasty (33.1% versus 11.9%, *P* = 0.001) and that of stent implantation (18.5% versus 6.0%, *P* = 0.017) were lower in the CC group. There was no significant difference between the two groups in anesthesia type, tandem lesions, vascular recanalization, embolism occurred during operation, sites of occlusion, time from onset to hospital, door to puncture time (DPT), puncture to recanalization time (PRT), and NIHSS score after EVT (*P* > 0.05).

**Table 2:** clinical characteristics stratified by cardiopulmonary comorbidities

Characteristics	All patients (*n* = 191)	NCC group (*n* = 124)	CC group (*n* = 67)	Test value	*P*-value
Intravenous thrombolysis, n(%)	75 (39.3%)	52 (41.9%)	23 (34.3%)	1.055^c^	0.304
General anesthesia, n(%)	109 (57.05%)	70 (56.5%)	39 (58.2%)	0.055^c^	0.815
Tandem lesion, n(%)	44 (23.0%)	32 (25.8%)	12 (17.9%)	1.530^c^	0.216
Times of thrombectomy, median(IQR)	2 (1,3)	1 (1,3)	2 (1,4)	2984^b^	0.001^*^
Balloon angioplasty, n(%)	49 (25.6%)	41 (33.1%)	8 (11.9%)	10.177^c^	0.001^*^
Stent implantation, n(%)	27 (14.1%)	23 (18.5%)	4 (6.0%)	5.670^c^	0.017^*^
Recanalization (mTICI 2b/3), n(%)	167 (87.4%)	110 (88.7%)	57 (85.1%)	0.523^c^	0.470
Intraoperative embolization, n(%)	24 (12.6%)	14 (12.9%)	10 (14.9%)	0.523^c^	0.470
Location of occlusion, n(%)				4.054^c^	0.132
Anterior circulation	162 (84.3%)	105 (84.7%)	57 (85.1%)		
Posterior circulation	27 (14.1%)	19 (15.3%)	8 (11.9%)		
Both anterior and posterior	2 (1%)	0 (0%)	2 (3.0%)		
ODT (min), mean ± SD	260.47 ± 225.07	269.95 ± 219.14	242.93 ± 236.33	0.794^a^	0.428
DPT (min), mean ± SD	91.04 ± 57.93	94.67 ± 65.03	84.33 ± 41.33	1.182^a^	0.239
PRT (min), mean ± SD	81.05 ± 57.39	78.43 ± 55.80	85.91 ± 60.34	-0.861^a^	0.390

*IQR* Interquartile range, *SD *Standard deviation, *mTICI* Modified thrombolysis in cerebral infarction, *NIHSS *The National Institutes of Health Stroke Scale, *ODT *Onset to door time, *DPT *Door to puncture time, *PRT *Puncture to recanalization time

^*^ for *P*<0.05

^a^ for t-value, ^b^ for Z-value, ^c^ for χ^2^ value

### Outcome

Efficacy and safety outcomes are presented in Table 3. The NIHSS at 24 h after EVT was higher in the CC group, and the difference was statistically significant [8(4,15.25) versus 12(7.5,18.5), *P* = 0.005]. At day 90, the good functional outcomes were achieved in 17 (25.4%) patients in the CC group, which was significantly less (*P* = 0. 000) than those in the NCC group. The mortality rate was numerically lower in the NCC group, but the difference was not significant (12.1% versus 14.9%, *P* = 0.580). Out of the total number of 25 mortality cases, 10 cases were in the CC group, including 5 cases of cerebral hernia or hemorrhage, 1 case of heart failure, 2 cases of myocardial infarction, 1 case of pulmonary infection and 1 case of renal failure, while the other 15 cases were in the NCC group, including 10 cases of cerebral hernia or hemorrhage, 1 case of gastrointestinal hemorrhage, 1 case of pulmonary embolism and 2 cases of respiratory failure. There was no difference in the proportion of any postoperative complications, including incidence of ISCHs, brain herniation and decompressive hemicraniectomy. To verify whether the age made the outcomes worse, we did a subgroup analysis between 22 elderly and 45 young patients in the CC group. The result showed that there was no significant difference in 90d good functional outcomes [26.7%(12/45) versus 29.4%(5/22), χ2 = 0.121, *P* = 0.728].

**Table 3:** Outcomes stratified by cardiopulmonary comorbidities

Characteristics	All patients (*n* = 191)	NCC group (*n* = 124)	CC group (*n* = 67)	Test value	*P*-value
90d mRS 0 ~ 2, n(%)	81 (42.4%)	64 (51.6%)	17 (25.4%)	12.262^c^	0.000^*^
NIHSS after EVT, mean ± SD	14.03 ± 9.51	13.51 ± 9.78	15.01 ± 8.97	-1.045^a^	0.297
NIHSS at 24 h, median (IQR)	10 (4,17.5)	8(4,15.25)	12 (7.5,18.5)	3210.50^b^	0.005^*^
Mortality, n(%)	25 (13.1%)	15 (12.1%)	10 (14.9%)	0.306^c^	0.580
Hemorrhage transformation, n(%)	30 (15.7%)	17 (13.7%)	13 (19.4%)	1.065^c^	0.302
Brain herniation, n(%)	19 (9.9%)	12 (9.7%)	7 (10.4%)	0.029^c^	0. 658
Decompressive hemicraniectomy, n(%)	10 (5.2%)	7 (5.6%)	3 (4.5%)	0.1200^c^	0.73

*IQR* Interquartile range, *SD* Standard deviation

^*^ for *P*<0.05

^a^ for t-value，^b^ for Z-value，^c^for χ^2^ value

### Prognosis analysis

**Fig. 2:**
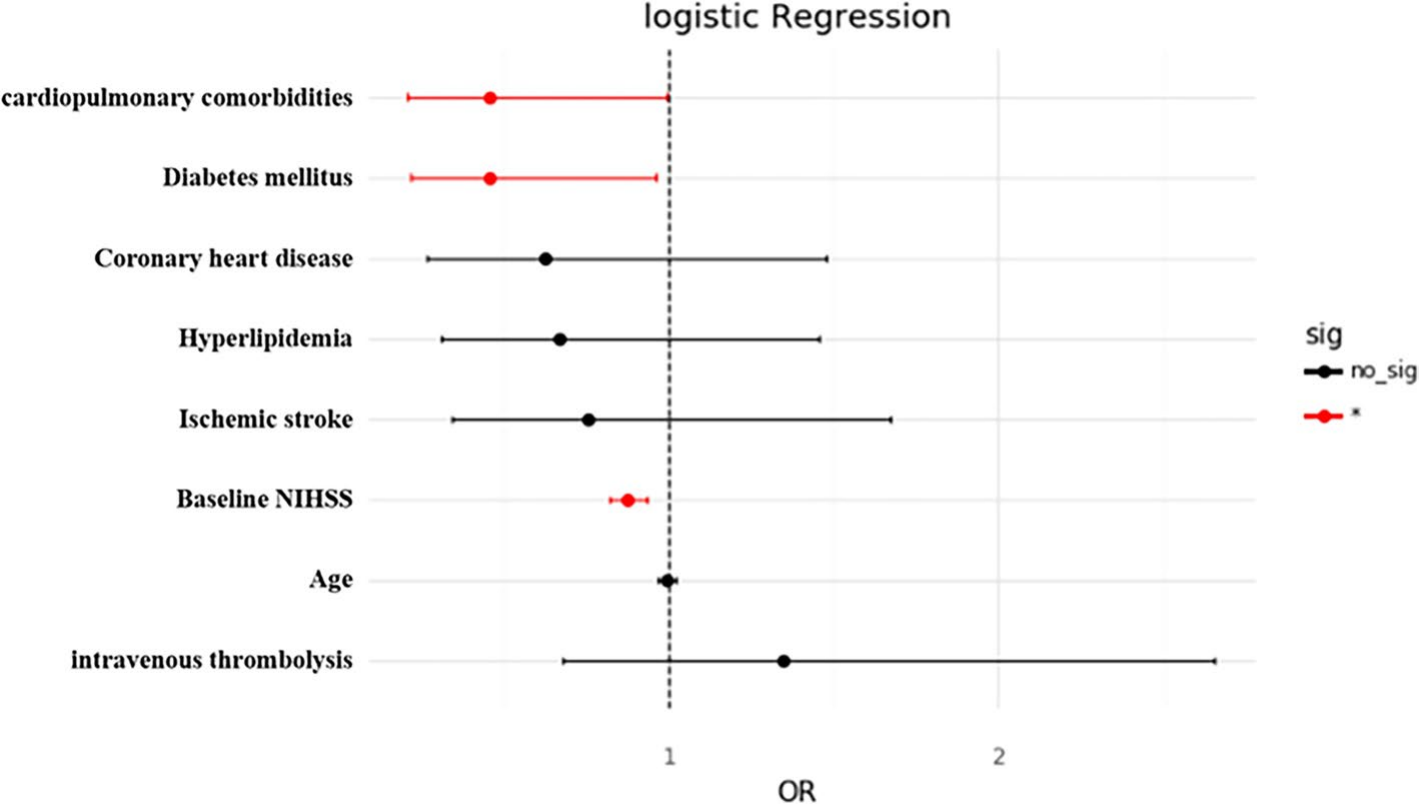
Forrest plot of multivariate analysis of 90-days outcomes. The red lines show the factor is significant in the logistic regression. The black lines show that there is no significance. ^*^ for *P* < 0.05, no_sig = no significant

**Fig. 3:**
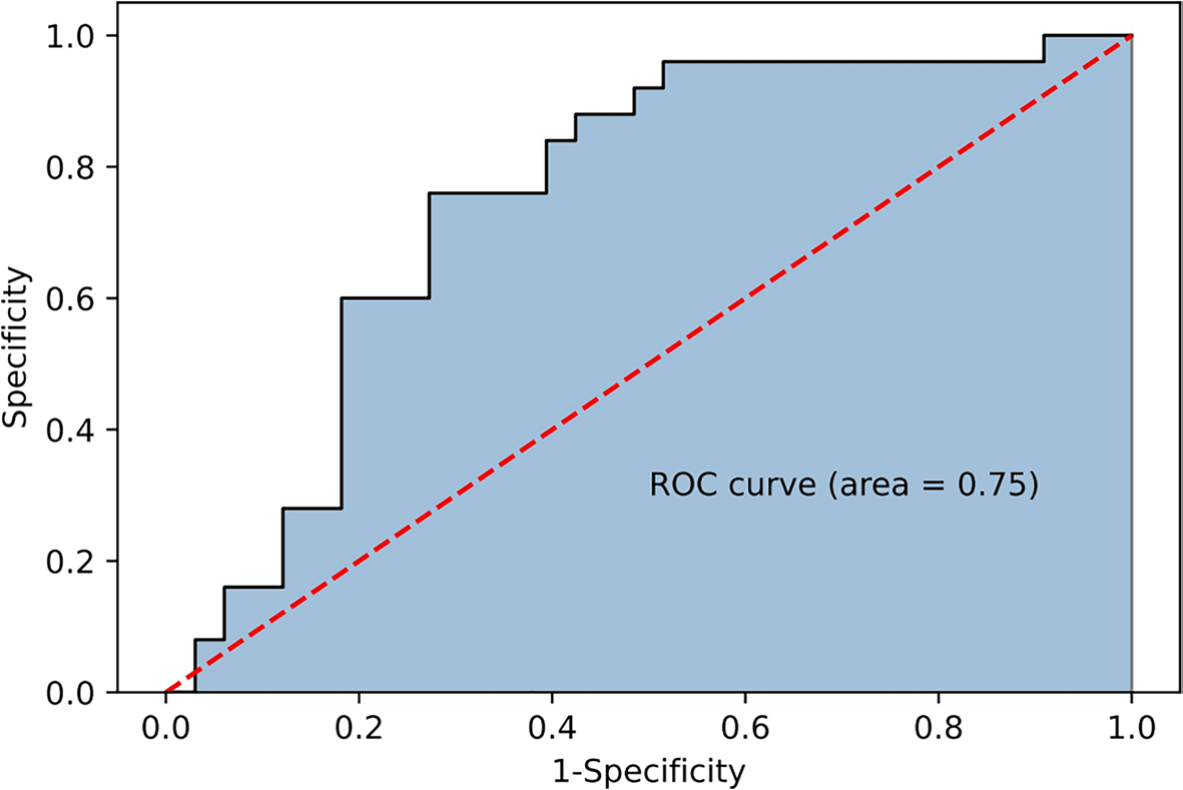
The receiver operating characteristic (ROC) curve of the logistic regression model for predicting outcomes. The x-axis meant the false-positive rate of the risk prediction

Then, a univariate analysis of all the patients is shown in Table 4. The good functional outcomes at day 90 (mRS score 0–2) were achieved in 81 (42.4%) patients. Among the variables that could be accessed to before EVT, the following was identified as predictors of a good outcome at 3 months (*P* < 0.1): Age, Ischemic stroke, Diabetes mellitus, Hyperlipidemia, Coronary heart disease, cardiopulmonary complication, intravenous thrombolysis, Baseline NIHSS. When predictors were selected for the logistic regression analysis (Table 5; Fig. 2), history of diabetes mellitus (OR = 0.459, 95%CI 0.219 to 0.960, *P* = 0.039), higher baseline NIHSS (OR = 0.880, 95%CI 0.820 to 0.935, *P* = 0.000) and existence of cardiopulmonary comorbidities (OR = 0.456, 95%CI 0.209 to 0.992, *P* = 0.048) were associated with a poor prognosis. The AUC of the logistic regression model was 0.75 (Fig. 3), the weighed F1 score was 0.73. It indicated that cardiopulmonary comorbidity was an independent risk factor for the prognosis.

**Table 4:** Univariate analysis of 90-days outcomes

Characteristics	All patients (*n* = 191)	Good outcome (*n* = 81)	Poor outcome (*n* = 110)	Test value	*P*-value
Male, n(%)	119 (62.3%)	54 (66.7%)	65 (59.1%)	1.140^c^	0.286
Medical history, n(%)					
Ischemic stroke	51 (26.7%)	16 (19.8%)	35 (31.8%)	3.470^c^	0.063
Diabetes mellitus	75 (39.3%)	22 (27.2%)	53 (48.2%)	8.644^c^	0.003^**^
Hypertension	129 (67.5%)	52 (64.2%)	77 (70.0%)	0.716^c^	0.397
Hyperlipidemia	74 (38.7%)	25 (30.9%)	49 (44.5%)	3.679^c^	0.055
Atrial fibrillation	92 (48.2%)	34 (42.0%)	58 (52.7%)	2.160^c^	0.142
Coronary heart disease	52 (27.2%)	13(16.0%)	39 (35.5%)	8.866^c^	0.003^**^
Smoking	37 (19.4%)	19 (23.5%)	18 (16.3%)	1.503^c^	0.220
Drinking	19 (9.9%)	10 (12.3%)	9 (8.1%)	0.903^c^	0.342
Cardiopulmonary comorbidities, n(%)	67 (35.1%)	17 (21.0%)	50 (45.5%)	12.262^c^	0.000^**^
Intravenous thrombolysis, n(%)	75 (39.3%)	38 (46.9%)	37 (33.6%)	3.448^c^	0.063^*^
General anesthesia, n(%)	109 (57.05%)	43 (53.1%)	66 (60.0%)	0.910^c^	0.340
Tandem lesion, n(%)	44 (23.0%)	17 (21.0%)	27 (24.5%)	0.333^c^	0.564
Balloon angioplasty, n(%)	49 (25.6%)	23 (28.4%)	26 (23.6%)	0.554^c^	0.457
Stent implantation, n(%)	27 (14.1%)	12 (14.8%)	14 (12.7%)	0.173^c^	0.678
Recanalization (mTICI 2b/3), n(%)	167 (87.4%)	79 (97.5%)	88 (80.0%)	13.049^c^	0.000^**^
Intraoperative embolization, n(%)	24 (12.6%)	7 (8.6%)	17 (15.5%)	1.971^c^	0.160
TOAST classification, n(%)				3.135^c^	0.209
Cardioembolism	70 (36.6%)	32 (39.5%)	38 (34.5%)		
Large-artery atherosclerosis	108 (56.5%)	41 (50.6%)	67 (60.9%)		
Other etiologies	13 (6.8%)	8 (9.9%)	5 (4.5%)		
Location of occlusion, n(%)				5.186^c^	0.075^*^
Anterior circulation	162 (84.8%)	74 (91.4%)	88 (80.0%)		
Posterior circulation	27 (14.1%)	7 (8.6%)	20 (18.2%)		
Both anterior and posterior	2 (1%)	0 (0%)	2 (1.8%)		
Age, mean ± SD	68.40 ± 13.68	65.70 ± 13.21	70.38 ± 13.74	-2.363^a^	0.019^**^
Baseline NIHSS, median(IQR)	16 (13, 21)	15 (12,17)	18.5 (14,23)	2715.00^b^	0.000^**^
Pre-stroke mRS, n(%)				9.4996^c^	0.087^*^
0	147 (77.0%)	70(86.4%)	77(70.0%)		
1	26 (13.6%)	9(11.1%)	17(15.5%)		
2	18 (9.4%)	2(2.5%)	16(14.5%)		
ODT (min), mean ± SD	260.47 ± 225.07	235.23 ± 203.35	279.08 ± 239.00	-1.335^a^	0.184
DPT (min), mean ± SD	91.04 ± 57.93	88.54 ± 63.60	92.88 ± 53.59	-0.504^a^	0.615
PRT (min), median(IQR)	67(36,109)	50(35,86)	78(39.5,130.75)	3353.00^b^	0.002^**^
ASPECTS, mean ± SD	8.52 ± 1.67	8.74 ± 1.53	8.35 ± 1.75	1.586^a^	0.114
Times of thrombectomy, median(IQR)	2 (1,3)	1 (1,2)	2 (1,4)	3046.00^b^	0.000^**^
NIHSS after EVT, median(IQR)	12(7,20)	7 (3,11)	17.5(12,22)	1310.50^b^	0.000^**^
NIHSS after EVT 24 h, median(IQR)	10 (4,17.5)	4 (2,6)	15(10.25,21.75)	637.50^b^	0.000^**^
Hemorrhage transformation	30(15.7%)	4(4.9%)	26(23.6%)	12.319^c^	0.000^**^
Brain herniation	19(9.9%)	0(0%)	19(17.3%)	15.536^c^	0.000^**^
Decompressive hemicraniectomy	10(5.2%)	0(0%)	10(9.1%)	7.770^c^	0.005^**^

*IQR* Interquartile range, *SD *Standard deviation, *mTICI *Modified thrombolysis in cerebral infarction, *NIHSS *The National Institutes of Health Stroke Scale, *ODT *Onset to door time, *DPT *Door to puncture time, *PRT *Puncture to recanalization time

^*^ for *P*<0.1, ^**^ for *P*<0.05

^a^ for t-value, ^b^ for Z-value, ^c^ for χ^2^ value

**Table 5:** Multivariate analysis of 90-days outcomes

Characteristics	Coefficient	*P*	OR	95%CI
Cardiopulmonary comorbidities	-0.7860	0.048^*^	0.456	(0.209, 0.992)
Diabetes mellitus	-0.7796	0.039^*^	0.459	(0.219, 0.960)
Coronary heart disease	-0.4699	0.284	0.625	(0.265, 1.477)
Hyperlipidemia	-0.4017	0.310	0.670	(0.308, 1.453)
Ischemic stroke	-0.2793	0.491	0.756	(0.342, 1.674)
Baseline NIHSS	-0.1331	0.000^*^	0.880	(0.820, 0.935)
Age	-0.0075	0.567	0.993	(0.967, 1.018)
Intravenous thrombolysis	0.2958	0.394	1.344	(0.680, 2.655)

^*^ for *P* < 0.05

## Discussion

We studied the characteristics and outcomes of thrombectomy in patients with cardiopulmonary diseases. Our retrospective study finds that baseline characteristics are not balanced, there are more female, older, higher proportion of atrial fibrillation and coronary heart disease, and smoking. These variables are risk factors of chronic lung [21] and cardiac [22, 23], as well as stroke. A number of retrospective studies have shown that patients with congestive heart failure is associated with atrial fibrillation (AF) [22, 24], which is consistent with the result in our study. Although ALVOS patients in the CC group have more AF, fewer stroke onset was caused by cardioembolism. It is speculated that long-term chronic cardiopulmonary dysfunction may result in an increase in the proportion of large-vessel atherosclerosis infarction.Chronic heart failure reduced cerebral blood flow and abnormal auto-regulation might result in impaired perfusion and arterial stiffness in the brain [25, 26]. Previous study showed poor pulmonary dysfunction could cause cerebral small vessel diseases [21, 27]. The lungs and heart are irrevocably linked in their oxygen and carbon dioxide transport functions [28]. Cardiopulmonary comorbidity reduces oxygen supply to the brain, which could lead to endothelial damage and accelerate the process of atherosclerosis, then aggravate the negative effect on pathogenesis of ischemia lesions.

Previous studies have shown that age is one of the factors of poor outcomes, but few have studied whether these populations comorbid with cardiopulmonary diseases. Our study shows that chronic cardiopulmonary comorbidity is a risk factor to the poor prognosis after EVT. Then, we performed a subgroup analysis of the CC group, predicting that the process of comorbidity leading to poor outcomes is not related to the age. On the contrary, the poorer cardiopulmonary function may account for the poor outcomes in elderly patients with ALVOS.

In terms of clinical characteristics between groups, patients with cardiopulmonary comorbidities were more likely to have higher mRS before stroke onset, while there was no difference in the baseline NIHSS score between the two groups. Therefore, we suspect that chronic cardiopulmonary comorbidity leads to slight neurological disability before EVLOS onset, instead of aggravating impairment at stroke onset. Besides, our results are comparable to the rate of recanalization achieved in large randomized trials with similar inclusion criteria [2, 29–31]. There were more intraoperative embolization and less rescue therapy including both balloon angioplasty and stent implantation in the CC group, indicating that treatment was more difficult. According to the recent studies, the composition of the thrombus may impact mechanical thrombectomy outcome [9]. Fibrin-rich thrombi have a less favorable outcome, mostly due to their increased stiffness and resistance to mechanical thrombectomy [8]. It has been acknowledged that one of the major hidden cause of occlusions refractory to modern mechanical thrombectomy procedures is underlying severe intracranial atherosclerotic stenosis (ICAS) [32, 33]. According to the Chinese Intracranial Atherosclerosis study, ICAS was the most common vascular lesion of stroke patients in China [34]. We could infer that chronic cardiopulmonary comorbidity may be accompanied by more ICAS before stroke onset and cause in situ thrombotic occlusion.

The poor efficacy outcomes of patients with cardiopulmonary dysfunction could be indicated at earliest at 24 h after EVT by the NIHSS and last until at day 90 by mRS, which was consistent with a study of MR CLEAN and IMS III trial [35]. Additionally, results of safety outcomes were no difference. The overall mortality rate was numerically lower in the NCC group, but the difference was not significant. Patients with chronic cardiopulmonary comorbidities were more likely to death from other system failure. In agreement of the previous findings that comorbidity is associated with higher mortality [11], we demonstrate that heart and respiratory comorbidities were associated with poor neurological recovery and could cause more death related to multisystem failure.

In terms of the relationship between cardiopulmonary comorbidity and outcomes at day 90, our study find that cardiopulmonary comorbidities can also be a predictor of poor outcomes. Newly developed clinical decision models no longer based on single patient characteristics [36]. Instead, multivariable regression models tend to join multiple baseline clinical and radiological characteristics, and conclude that large variations in treatment can benefit patients. However, there is still no consensus in prognosis model on predictor factors selection, for it is uncertain about treatment benefit in specific subgroups. In our multivariable regression analysis, history of diabetes mellitus, higher baseline NIHSS and existence of cardiopulmonary comorbidity are associated with a poor prognosis. Thus, cardiopulmonary comorbidity is an independent risk factors to predict outcomes before EVT and assist clinical decision making.

Compared to the previous studies [31], our result firstly reports that cardiopulmonary comorbidity could lead to a worse ending. We have focused on cardiac and pulmonary dysfunction, which are not traditional factors of the vascular diseases, and those patients are generally underrepresented in randomized trial [7, 37]. Previously, the effect of cardiac and pulmonary dysfunction on cerebral arteries were studied separately, but few noticed the combined effect of them on patients with ELVOS and undergoing emergency thrombectomy. Patients with chronic heart failure and respiratory failure commonly have the diagnostic symptom of exertional dyspneoea varies in intensity [10, 38] and lead to more intensive condition after stroke. Early identification and timely treatment can reduce the mortality in neurological intensive care units to a certain extent.

Our study had limitations. First, there were the general limitations of the single-center and small-sample retrospective study design, which selection and information bias cannot be ruled out. Second, the revascularization techniques were not uniform and scattered. Third, process that comorbidity affected AIS prognosis were complicated, especially when the subjects were elderly people and our baseline characteristics were unbalanced in our study. Therefore, multi-center randomized controlled studies are needed for further validation.

## Conclusion

Based on our results, we conclude that cardiopulmonary comorbidities are important risk factors that have been overlooked for a long time. Endovascular thrombectomy was safe in ALVOS patients with chronic cardiopulmonary diseases. However, the unfavorable outcomes were achieved in such patients. The effect of cardiopulmonary morbidity on ALVOS is mixed. Moreover, cardiopulmonary comorbidity was a powerful predictor of worse clinical outcomes and should be considered when neurologists make individualising selection of patients for EVT.

### Supplementary Information

Supplementary Material 1: S1 Text. STROBE checklist. STROBE, Strengthening the Reporting of Observational Studies in Epidemiology.

## Data Availability

Restrictions apply to the availability of these data and they are not publicly available. However, data are available from the corresponding author upon reasonable request and with the permission of the institution.
